# Rapid Assay for the Therapeutic Drug Monitoring of Edoxaban

**DOI:** 10.3390/biom12040590

**Published:** 2022-04-17

**Authors:** Md Abdur Rashid, Saiqa Muneer, Yahya Alhamhoom, Nazrul Islam

**Affiliations:** 1 Department of Pharmaceutics, College of Pharmacy, King Khalid University, Guraiger, Abha 62529, Saudi Arabia; ysalhamhoom@kku.edu.sa; 2 School of Chemistry and Physics, Queensland University of Technology, Brisbane, QLD 4000, Australia; saiqa.muneer@hdr.qut.edu.au; 3 Pharmacy Discipline, Faculty of Health, School of Clinical Sciences, Queensland University of Technology, Brisbane, QLD 4000, Australia; nazrul.islam@qut.edu.au

**Keywords:** edoxaban, therapeutic drug monitoring, surface enhanced Raman spectroscopy, pulmonary embolism

## Abstract

Edoxaban is a direct oral anticoagulant (DOAC) that has been recently indicated for the treatment of pulmonary embolism (PE) in SARS-CoV-2 infections. Due to its pharmacokinetic variability and a narrow therapeutic index, the safe administration of the drug requires its therapeutic drug monitoring (TDM) in patients receiving the treatment. In this work, we present a label-free method for the TDM of edoxaban by surface enhanced Raman spectroscopy (SERS). The new method utilises the thiol chemistry of the drug to chemisorb its molecules onto a highly sensitive SERS substrate. This leads to the formation of efficient hotspots and a strong signal enhancement of the drug Raman bands, thus negating the need for a Raman reporter for its SERS quantification. The standard samples were run with a concentration range of 1.4 × 10^−4^ M to 10^−12^ M using a mobile phase comprising of methanol/acetonitrile (85:15 v/v) at 291 nm followed by the good linearity of R^2^ = 0.997. The lowest limit of quantification (LOQ) by the SERS method was experimentally determined to be 10^−12^ M, whereas LOQ for HPLC-UV was 4.5 × 10^−7^ M, respectively. The new method was used directly and in a simple HPLC-SERS assembly to detect the drug in aqueous solutions and in spiked human blood plasma down to 1 pM. Therefore, the SERS method has strong potential for the rapid screening of the drug at pathology labs and points of care.

## 1. Introduction

SARS-CoV-2 infection can lead to pulmonary embolism (PE) and the strong activation of blood coagulation processes in patients, thus leading to excessive blood clotting [1,2]. The treatment of these side effects requires the administration of anticoagulant drugs to prevent blood clotting [3]. Edoxaban is a direct oral anticoagulant (DOAC) that has been recently demonstrated as an effective treatment to inhibit IIa or Xa factors in SARS-CoV-2 patients and reduce the potential for excessive blood [1,4]. Similar to other DOACs, edoxaban has large pharmacokinetic inter-individual variability and a narrow therapeutic index [5,6,7,8,9]. Therefore, there is a need to monitor the drug concentration in patients to avoid potential adverse side effects/reactions [8].

The quantification of edoxaban in human blood plasma has been demonstrated by liquid chromatography–diode array detector (LC-DAD) and mass spectrometry (LC-MS) methods, chromogenic assay, and clotting assay [9,10,11,12,13,14]. However, the LC-MS method is time consuming and requires complex sample preparation steps and skilled personnel to carry out the analysis and interpret the MS results [15,16] Therefore, clotting assays have been more widely used for the screening of edoxaban in patient blood. However, this method cannot be used for the accurate quantification of the drug concentration in the sample [17,18].

Surface-enhanced Raman spectroscopy (SERS) is a highly sensitive analytical tool that can be used for the rapid screening of drugs in biological fluid [19,20,21]. In SERS, the Raman emission of analyte molecules is enhanced by several orders of magnitude when they are adsorbed onto the nanostructures of a noble metal and excited by incident light (excitation beam). The enhancement is due to the interaction between the surface plasmons of the metallic nanostructures and the incident light which leads to a strong electromagnetic field that boosts the Raman signal of the excited analyte molecules (electromagnetic field enhancement). In addition, the formation of a charge–transfer complex between the adsorbed analyte molecules and the metallic nanostructures of the SERS sensor reduces the energy gap between the ground and excited states of the analyte–metal system and facilitates the flow of electrons between the electronic states, thus leading to a further enhancement of the analyte Raman signal (chemical enhancement) [22].

Many materials have been used to fabricate sensitive SERS sensors. These materials include noble metals (gold, silver), metal oxides (ZnO, SnO_2_, TiO_2_), and metal–organic frameworks [23,24]. Unlike silver, the gold nanostructures are inert towards oxidation processes that can compromise their SERS activity. Therefore, gold nanostructured substrates are frequently used for the screening of bioactive molecules due to their high SERS enhancement, resistance to oxidation, and long shelf life.

Herein, we report a label-free SERS assay for the determination of edoxaban in human blood plasma without the need for a Raman reporter. The drug chemisorbs, through its sulphur moiety, to the gold nanostructures of a silicon nanopillar substrate, thus leading the nanopillars to lean towards each other with the drug molecules trapped between them. This surface modification causes the formation of efficient hotspots where the trapped drug molecules experience a strong electromagnetic field and large enhancement of their Raman signal. Additional chemical enhancement also occurs due to the formation of a charge–transfer complex between the gold nanostructures and the drug. Using the new SERS assay, either directly or in an HPLC-SERS assembly, edoxabane was quantified in spiked human blood plasma down to 1 pM. Utilising the new SERS method in an HPLC-SERS assembly does not only provide a highly sensitive quantification of the drug but also its fingerprint identification without the use of a sophisticated and expensive detector such as the mass detector.

## 2. Material and Methods

### 2.1. Materials

Gold-coated silicon nanopillar substrates were purchased from Silmeco (Copenhagen, Denmark). The HPLC grade acetonitrile, formic acid, pharmaceutical grade edoxaban, and human blood plasma were purchased from SIGMA ALDRICH (St. Louis, MO, USA) and used under human research ethics exemption given by Queensland University of Technology (number 1800001209). All aqueous solutions were prepared using deionised water (18.2 MΩ cm). Dimethyl sulfoxide (99.9%) was purchased from Cambridge Isotope Laboratories, Tewksbury, MA 01876, United States.

### 2.2. Instrumentation 

Handheld Raman spectrometer (ID Raman mini-2.0, Ocean Optics, Inc, Dunedin, FL34698, United States; spectral resolution 12 cm^−1^) was used for the SERS measurements. The spectrometer was operated in Orbital Raster Scanning (ORS) mode. The measurements were carried out in the wavelength range of 400 cm^−1^–1800 cm^−1^. Sample excitation was carried out using a 785 nm laser source. The laser power at the sample was 5 mW. The Raman spectra were collected using an acquisition time of 1 s per measurement (10 accumulations). The instrument software algorithm (Ocean View Spectroscopy 1.5.07, NY-FluxData Product Line, NY 14607, United States) was operated to automatically correct the background noise and fluorescence in the SERS measurements. 

Liquid chromatography was carried out using Agilent HPLC 1100 chromatograph (Agilent, Santa Clara, CA 95051,United States). A diode array was utilised as a detector and set at 291 nm to monitor the separation and retention time of the drug. Guard and analytical UHPLC Column Porshell 120, EC-C18, 4.6 × 250 mm, 4 µm and HPLC Column Poroshell 120 EC-C18, 2.7 µm, 4.6 × 150 mm respectively were purchased from Agilent, Santa Clara, CA 95051, United States and used for the separation of edoxaban.

### 2.3. Preparation of Edoxaban Standard Solutions

Edoxaban stock solution (1 mg/mL) was prepared by dissolving 1 mg of the drug in 1 mL of DMSO. A 100 µg/mL aliquot of the drug stock solution was mixed with 10 mL of deionised water to prepare a 1.4 × 10^−4^ M edoxaban standard solution. Standard solutions of the drug were prepared in the concentration range of 1.4 × 10^−4^ M to 10^−12^ M by serial dilution using a DMSO: water solvent (1:10 *v*/*v*).

### 2.4. SERS Quantification of Edoxaban

Two hundred microliters of the edoxaban solutions in the concentration range of 1.4 × 10^−4^ M–10^−12^ M (0.548 µg/mL to 0.548 pg/mL) was loaded onto the gold-coated silicon nanopillar substrates and left to stand for 15 min. The substrates were then rinsed three times with DMSO: water solvent (1:10 *v*/*v*), dried under a gentle stream of nitrogen gas, and the SERS measurements were carried out using the handheld Raman spectrometer (*n* = 3). A calibration plot for edoxaban was developed by plotting the intensity of the Raman band at 1436 cm^−1^ against log the concentration of the drug.

### 2.5. Reproducibility of SERS Measurements

To demonstrate the reproducibility of the SERS method, a 200 µL aliquot of 1 × 10^−6^ M edoxaban standard solution was loaded onto three independent SERS substrates and allowed to stand for 15 min. The substrates were then washed three times with DMSO: water solvent (1:10 *v*/*v*), dried under nitrogen gas then screened by SERS (*n* = 3). The relative standard deviation (RSD) between the SERS measurements was calculated by monitoring the change in the Raman signal intensity at 1436 cm^−1^.

### 2.6. Positive and Negative Control Tests

Positive and negative control samples were screened by the SERS method. For a negative control test, blank human blood plasma was loaded onto gold-coated silicon nanopillar substrate for 15 min, then washed three times with DMSO: water solvent (1:10 *v*/*v*). The substrate was then dried under a gentle stream of nitrogen gas and screened by the handheld Raman spectrometer (*n* = 3). For a positive control test, the human blood plasma was spiked with edoxaban to the final concentration of 1 × 10^−6^ M, loaded onto a gold-coated silicon nanopillar substrate and the above procedures were repeated. The dry substrate was screened by the handheld Raman spectrometer (*n* = 3).

### 2.7. Cross Validation by HPLC-UV Method

The new SERS measurements were cross-validated using HPLC-UV [25]. The mobile phase was composed of methanol and acetonitrile (85:15 *v*/*v*), the flow rate of the mobile phase was adjusted to 1.00 mL/min and the total run time of the chromatographic separation was 10 min. The injection volume of the edoxaban standard solutions was 20 µL. A diode array detector at 291 nm was used to monitor the chromatographic separation of the drug. A calibration plot was established by plotting the area under the peak at 2.68 min against the concentration of the drug in the concentration range of 0.25 µg/mL–100 µg/mL (Figure S1).

### 2.8. Determination of Edoxaban in Spiked Blood Plasma

HPLC-SERS assembly was used to determine edoxaban in spiked blood plasma [26]. Human blood plasma was spiked with edoxaban solution to the final concentration of 1.4 × 10^−4^ M. Twenty microlitres of the spiked human blood plasma were injected onto the HPLC column and the chromatographic separation was carried out. The eluate at 2.68 min was deposited onto a gold-coated silicon nanopillar sensor and screened by SERS.

## 3. Results and Discussion

### 3.1. SERS Measurement of Edoxaban

**Figure 1:**
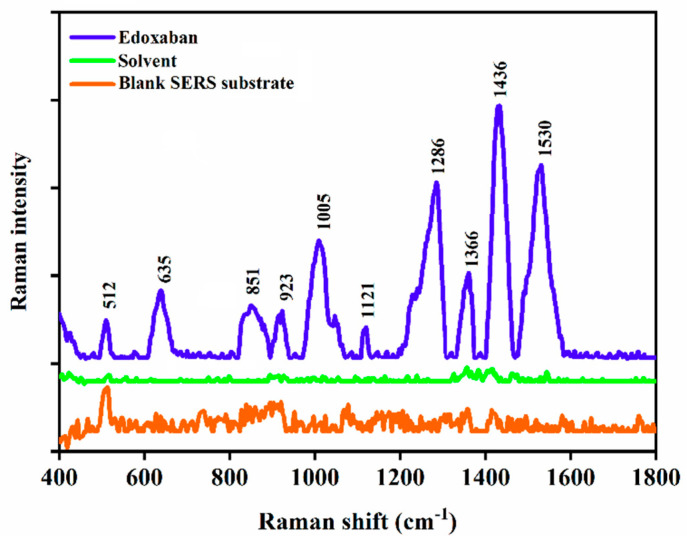
SERS spectrum of edoxaban; in DMSO: water (1:10 *v*/*v*) solvent; and bare gold-coated silicon nanopillar SERS substrate.

Two- and three-dimension nanostructured substrates have been demonstrated in the literature as SERS sensors for the detection of numerous analytes. 2D materials have the advantages of ease of synthesis, a large SERS active surface area and good biocompatibility [27]. On the other hand, 3D substrates have the advantage of maximising the interaction between the analyte, the substrate, and the excitation light, thus providing highly sensitive SERS measurements [28]. Therefore, in this work, we used a 3D gold-coated silicon nanopillar substrate to acquire highly sensitive SERS measurements of edoxaban. The SEM image of the gold-coated silicon nanopillar SERS sensor is depicted in Figure S2. As indicated by the figure, the gold nanostructures on the silicon pillars are separated by capillary-like gaps [29]. When the edoxaban molecules diffuse within these gaps, the sulphur moiety within the molecular structure of the drug chemisorbs onto the gold surface via Au–S bonds to form a charge–transfer complex [30]. As indicated in Figure 1, the SERS measurement of edoxaban showed Raman bands at 512 cm^−1^ and 635 cm^−1^ that can be attributed to S–S and C–S bonds, respectively. This causes the flexible gold-coated nanopillars to lean inwards towards each other [29]. Therefore, the gaps between the gold nanostructures decrease and their surface plasmons overlap to form hot spots that cause a strong electromagnetic field and chemical enhancements to the SERS measurement of the trapped drug when it is probed by the handheld Raman spectrometer [31]. As indicated in Figure 1, the SERS measurement of edoxaban showed Raman bands at 512 cm^−1^ and 635 cm^−1^ that can be attributed to the S–S and C–S bonds, respectively [32,33]. The Raman band at 1005 cm^−1^ can be attributed to the vibration mode of the benzene residues in the molecular structure of edoxaban (Figure S3) [31,32]. The Raman bands at 1436 cm^−1^, 1286 cm^−1^, 1336 cm^−1^, and 1536 cm^−1^ can be attributed to N–O, C–N and C–O stretching modes, respectively [33,34].

### 3.2. SERS Quantification of Edoxaban and Cross-Validation against HPLC-UV Method

**Figure 2:**
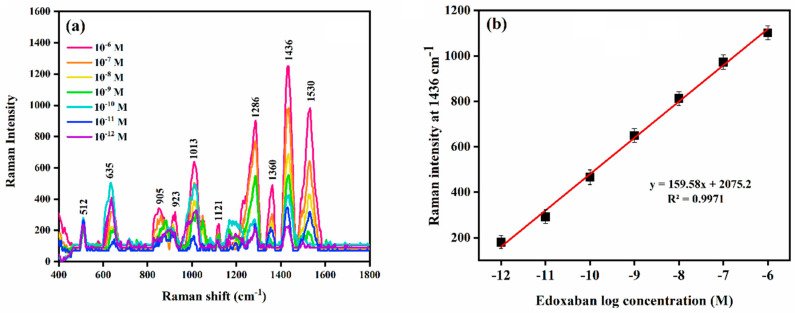
(**a**) SERS measurements of edoxaban at different concentrations; and (**b**) SERS calibration plot in the concentration range of 1.4 × 10^−4^ M to 10^−12^ M.

The edoxaban Raman band at 1436 cm^−1^ was found to change monotonically with the concentration of the drug (Figure 2a). Therefore, it was used to quantify the drug concentration by SERS. The Raman band intensity at 1436 cm^−1^ was plotted against log the concentration of edoxaban and a linear relationship was found in the concentration range 1.4 × 10^−4^ M to 10^−12^ M and followed the regression equation y = 159.58x + 2075.2 (R^2^ = 0.997) (Figure 2b). The lowest limit of quantification (LOQ) by the SERS method was experimentally determined to be 10^−12^ M.

HPLC-UV was used to re-screen the drug in aqueous standard solutions and cross-validate the SERS method. The retention time of the drug was determined to be 2.68 min (Figure S1a), and the LOQ of the method was 4.5 × 10^−7^ M (Figure S1b). We utilised the SERS and HPLC methods for the screening of edoxaban in spiked human blood plasma. The concentration of the drug in the sample was found to be 1.34 × 10^−4^ M by the SERS method and 1.4 × 10^−4^ M by the HPLC method. Therefore, the % agreement between the two methods was found to be 95.7%.

### 3.3. Selectivity of the SERS Method

**Figure 3:**
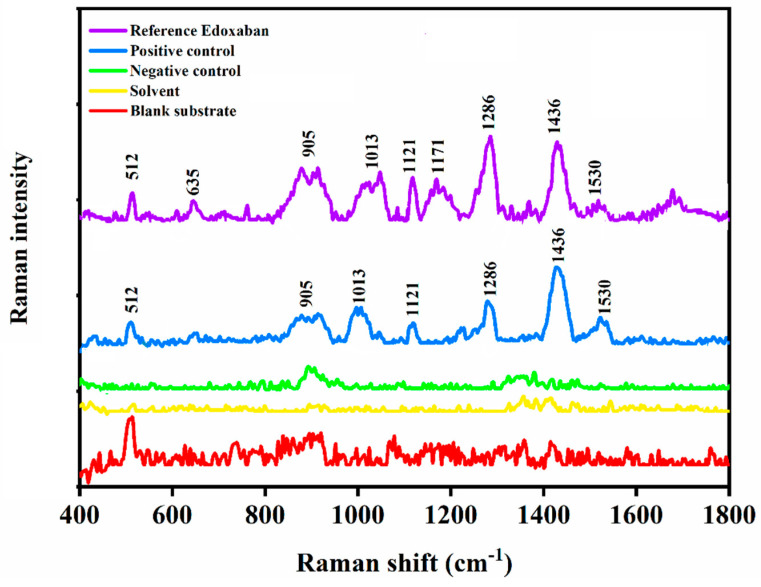
SERS spectrum of edoxaban (purple line), positive control sample (blue line), negative control sample (green line), DMSO: water (1:10 *v*/*v*) solvent (yellow line), and the gold-coated silicon nanopillar substrate (red line). All measurements were carried out on gold-coated silicon nanopillar substrates.

The selectivity of SERS method was demonstrated by screening positive and negative control samples. As depicted in Figure 3, the SERS measurement of the negative control sample did not show the diagnostic Raman bands of edoxaban. However, the SERS measurement of the positive control sample, showed the Raman fingerprint of edoxaban, thus confirming the direct detection of the drug in the spiked blood plasma matrix. This result can be attributed to the chemisorption of the drug molecule on the surface of the sensor through its sulphur moiety and the formation of strong Au–S bonds that caused the drug molecule to persist within the narrow capillary channels of the SERS substrate and not to wash away by the DMSO: water solvent during the rinsing process [30]. These results confirm the potential of the new SERS method for the direct and rapid determination of edoxaban, especially for high-risk patients such as individual suffering from serious SARS-CoV-2 infection side effects and receiving the drug as part of their therapy.

### 3.4. Reproducibility of the SERS Measurements

**Figure 4:**
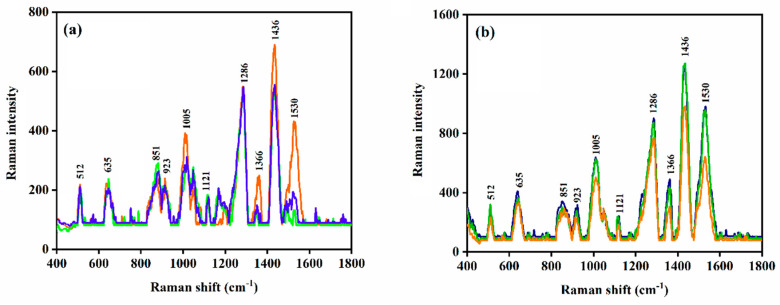
Reproducibility of SERS measurements at 1436 cm^−1^: (**a**) three measurements on a single substrate; and (**b**) three measurements on three independent substrates. All measurements were carried out on gold-coated silicon nanopillar substrates.

To demonstrate the reproducibility of the SERS method, the edoxaban standard solution was loaded onto a gold-coated silicon nanopillar sensor and the SERS measurement was repeated three times (Figure 4a). The RSD in the Raman signal intensity at 1436 cm^−1^ was found to be 2.87%. The drug standard was also loaded onto three gold-coated silicon nanopillar substrates and SERS measurements were carried out (*n* = 3), (Figure 4b). The RSD in the Raman signal intensity at 1436 cm^−1^ was 6.73%. The low RSD within the SERS measurements indicate the reproducibility of the SERS method and its potential for the TDM of edoxaban in patients.

### 3.5. HPLC-SERS Detection of Edoxaban in Spiked Human Blood Plasma

**Figure 5:**
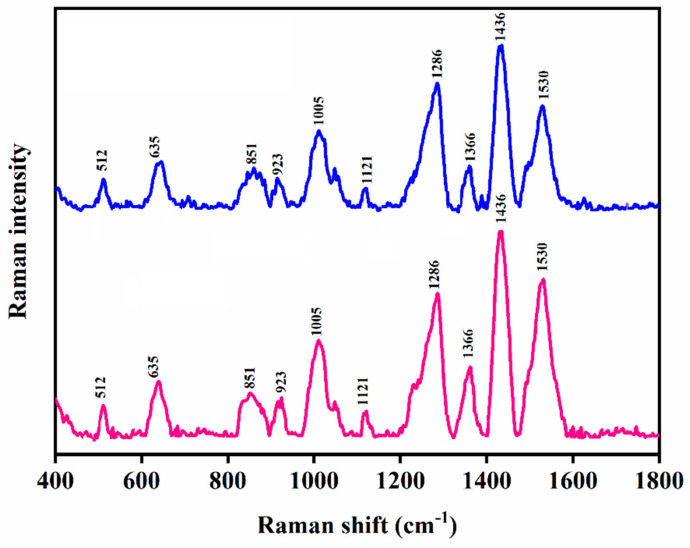
SERS measurements of the edoxaban standard (blue spectrum) and edoxaban eluate at 2.68 min after chromatographic separation (red spectrum).

The SERS sensor was also utilised in an HPLC-SERS assembly for the simultaneous separation and fingerprint identification edoxaban in spiked blood plasma (concentration = 1.4 × 10^−4^ M) (Figure S4) [26]. The chromatographic separation of the drug was carried out using the operating parameters of the HPLC-UV method and the eluate at 2.68 min was deposited onto a gold-coated substrate and screened by the handheld Raman spectrometer. As indicated by Figure 5, the Raman spectrum of the eluate at the drug retention time was similar to that of edoxaban. The concentration of the drug in the sample was quantified by SERS and found to be 1.32 × 10^−4^ M. Therefore, the % recovery of the HPLC-SERS method was 94.28%. This result indicates the potential for the SERS sensor to replace the UV and mass detectors for the combined fingerprint identification and quantification of edoxaban in human blood plasma.

### 3.6. Detection of Edoxaban Using Low-Cost SERS Substrate

**Figure 6:**
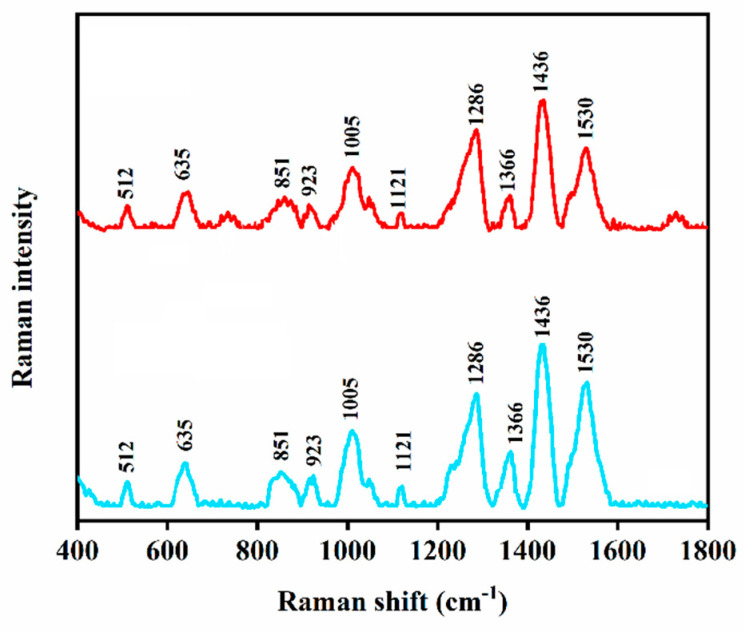
SERS measurement of edoxaban using silicon nanopillar substrate (blue spectrum) and using gold-coated copper oxide substrate (red spectrum).

To reduce the cost of the SERS method, we replaced the gold-coated silicon nanopillar substrate with a low-cost gold-coated copper oxide substrate and used it to screen 10^−6^ M edoxaban. As indicated by Figure 6, the Raman spectrum of edoxaban that is recorded by the gold-coated copper oxide substrate was in good agreement with that acquired using the gold-coated silicon nanopillar substrate. The gold-coated copper oxide substate gold-coated copper oxide substrate is fabricated using low-cost ion itching and sputtering methods [35,36]. Therefore, it can be utilised as a low-cost substrate to reduce the cost of the TDM of edoxaban by SERS.

## 4. Conclusions

A simple and rapid SERS assay was developed for the TDM of edoxaban in patients. The method showed high sensitivity when compared to the HPLC-UV method and reproducibility that led to a low RSD in the SERS measurements on independent substrates. The SERS method was utilised for the screening of edoxaban in spiked human blood plasma, and cross-validated against HPLC-UV. Therefore, the new method has strong potential for the TDM of edoxaban in SARS-CoV-2 patients receiving anticoagulant medication.

## Data Availability

Not Applicable.

## Supplementary Materials

The following supporting information can be downloaded at: https://www.mdpi.com/article/10.3390/biom12040590/s1. Figure S1: (a) Chromatographic separation of edoxaban by HPLC-UV method (b) calibration plot of the drug at retention time of 2.68 min. Figure S2: SEM image of the gold coated silicon nanopillar substrate. Figure S3: Chemical structure of edoxaban. Figure S4: Chromatographic separation of edoxaban by HPLC-SERS method (retention time of the drug = 2.68 min).

## References

[1] Al-Horani R.A. (2020). Potential Therapeutic Roles for Direct Factor Xa Inhibitors in Coronavirus Infections. Am. J. Cardiovasc. Drugs.

[2] Langella V., Bottino R., Asti A., Maresca G., Di Palma G., Pomponi D., Sassone C., Imbalzano E., Russo V. (2021). Edoxaban for the treatment of pulmonary embolism in hospitalized COVID-19 patients. Expert Rev. Clin. Pharmacol..

[3] Melo H.A.B.d., Faria S.S., Nascimento G.P.d., Santos I.d.O., Kobinger G.P., Magalhães K.G. (2021). The use of the anticoagulant heparin and corticosteroid dexamethasone as prominent treatments for COVID-19. Front. Med..

[4] Fenger-Eriksen C., Münster A.-M.B., Grove E. (2014). New oral anticoagulants: Clinical indications, monitoring and treatment of acute bleeding complications. Acta Anaesthesiol. Scand..

[5] Kanuri S.H., Kreutz R.P. (2019). Pharmacogenomics of Novel Direct Oral Anticoagulants: Newly Identified Genes and Genetic Variants. J. Pers. Med..

[6] Dunois C. (2021). Laboratory Monitoring of Direct Oral Anticoagulants (DOACs). Biomed..

[7] Wieland E., Shipkova M. (2019). Pharmacokinetic and Pharmacodynamic Drug Monitoring of Direct-Acting Oral Anticoagulants: Where Do We Stand?. Ther. Drug Monit..

[8] Scridon A., Serban R.C. (2016). Laboratory monitoring: A turning point in the use of new oral anticoagulants. Ther. Drug Monit..

[9] Zhang W.-L., Lou D., Zhang D.-T., Zhang Y., Huang H.-J. (2016). Determination of rivaroxaban, apixaban and edoxaban in rat plasma by UPLC–MS/MS method. J. Thromb. Thrombolysis.

[10] Gouveia F., Bicker J., Santos J., Rocha M., Alves G., Falcão A., Fortuna A. (2020). Development, validation and application of a new HPLC-DAD method for simultaneous quantification of apixaban, dabigatran, edoxaban and rivaroxaban in human plasma. J. Pharm. Biomed..

[11] Zhao Y., Couchman L., Kipper K., Arya R., Patel J.P. (2020). A UHPLC-MS/MS method to simultaneously quantify apixaban, edoxaban and rivaroxaban in human plasma and breast milk: For emerging lactation studies. J. Chromatogr. B.

[12] Wiesen M.H., Blaich C., Streichert T., Michels G., Müller C. (2017). Paramagnetic micro-particles as a tool for rapid quantification of apixaban, dabigatran, edoxaban and rivaroxaban in human plasma by UHPLC-MS/MS. Clin. Chem. Lab. Med. CCLM.

[13] He L., Kochan J., Lin M., Vandell A., Brown K., Depasse F. (2017). Determination of edoxaban equivalent concentrations in human plasma by an automated anti-factor Xa chromogenic assay. Thromb. Res..

[14] Hanada K., Matsumoto S.-I., Shibata S., Matsubara H., Tsukimura Y., Takahashi H. (2018). A quantitative LC/MSMS method for determination of edoxaban, a Xa inhibitor and its pharmacokinetic application in patients after total knee arthroplasty. Biomed. Chromatogr..

[15] Lindahl S., Dyrkorn R., Spigset O., Hegstad S. (2018). Quantification of apixaban, dabigatran, edoxaban, and rivaroxaban in human serum by UHPLC-MS/MS—Method development, validation, and application. Ther. Drug Monit..

[16] Bylda C., Thiele R., Kobold U., Volmer D.A. (2014). Recent advances in sample preparation techniques to overcome difficulties encountered during quantitative analysis of small molecules from biofluids using LC-MS/MS. Analyst.

[17] Berger A.G., Restaino S.M., White I.M. (2017). Vertical-flow paper SERS system for therapeutic drug monitoring of flucytosine in serum. Anal. Chim. Acta.

[18] Ashbee H.R., Barnes R.A., Johnson E.M., Richardson M.D., Gorton R., Hope W.W. (2013). Therapeutic drug monitoring (TDM) of antifungal agents: Guidelines from the British Society for Medical Mycology. J. Antimicrob. Chemother..

[19] Turzhitsky V., Zhang L., Horowitz G.L., Vitkin E., Khan U., Zakharov Y., Qiu L., Itzkan I., Perelman L.T. (2018). Picoanalysis of Drugs in Biofluids with Quantitative Label-Free Surface-Enhanced Raman Spectroscopy. Small.

[20] Szaniawska A., Kudelski A. (2021). Applications of Surface-Enhanced Raman Scattering in Biochemical and Medical Analysis. Front. Chem..

[21] Sultan M.A., El-Alamin M.M.A., Wark A.W., Azab M.M. (2019). Detection and quantification of warfarin in pharmaceutical dosage form and in spiked human plasma using surface enhanced Raman scattering. Spectrochim. Acta Part A Mol. Biomol. Spectrosc..

[22] Muneer S., Sarfo D.K., Ayoko G.A., Islam N., Izake E.L. (2020). Gold-Deposited Nickel Foam as Recyclable Plasmonic Sensor for Therapeutic Drug Monitoring in Blood by Surface-Enhanced Raman Spectroscopy. Nanomaterials.

[23] Gushiken N.K., Paganoto G.T., Temperini M.L.A., Teixeira F.S., Salvadori M.C. (2020). Substrate for Surface-Enhanced Raman Spectroscopy Formed by Gold Nanoparticles Buried in Poly(methyl methacrylate). ACS Omega.

[24] Rashid A., Muneer S., Mendhi J., Sabuj M.Z.R., Alhamhoom Y., Xiao Y., Wang T., Izake E.L., Islam N. (2021). Inhaled Edoxaban dry powder inhaler formulations: Development, characterization and their effects on the coagulopathy associated with COVID-19 infection. Int. J. Pharm..

[25] Sankar P.R., Eswarudu M., Krishna P.S., Srikanth D., Babu P.S., Rohith N. (2021). Novel validated RP-HPLC method for determination of edoxaban tosylate monohydrate in bulk and its pharmaceutical dosage form. J. Pharm. Sci..

[26] Subaihi A., Trivedi D.K., Hollywood K.A., Bluett J., Xu Y., Muhamadali H., Ellis D.I., Goodacre R. (2017). Quantitative Online Liquid Chromatography–Surface-Enhanced Raman Scattering (LC-SERS) of Methotrexate and its Major Metabolites. Anal. Chem..

[27] Chen M., Liu D., Du X., Lo K.H., Wang S., Zhou B., Pan H. (2020). 2D materials: Excellent substrates for surface-enhanced Raman scattering (SERS) in chemical sensing and biosensing. TrAC Trends Anal. Chem..

[28] Chen Y.-C., Hong S.-W., Wu H.-H., Wang Y.-L., Chen Y.-F. (2021). Rapid Formation of Nanoclusters for Detection of Drugs in Urine Using Surface-Enhanced Raman Spectroscopy. Nanomaterials.

[29] Ashley J., Wu K., Hansen M.F., Schmidt M.S., Boisen A., Sun Y. (2017). Quantitative Detection of Trace Level Cloxacillin in Food Samples Using Magnetic Molecularly Imprinted Polymer Extraction and Surface-Enhanced Raman Spectroscopy Nanopillars. Anal. Chem..

[30] Häkkinen H. (2021). The gold–sulfur interface at the nanoscale. Nat. Chem..

[31] Rae S.I., Khan I. (2010). Surface enhanced Raman spectroscopy (SERS) sensors for gas analysis. Analyst.

[32] Das G., Mecarini F., Gentile F., De Angelis F., Kumar H.M., Candeloro P., Liberale C., Cuda G., Di Fabrizio E. (2009). Nano-patterned SERS substrate: Application for protein analysis vs. temperature. Biosens. Bioelectron..

[33] Göksel Y., Zor K., Rindzevicius T., Als-Nielsen B.E.T., Schmiegelow K., Boisen A. (2021). Quantification of Methotrexate in Human Serum Using Surface-Enhanced Raman Scattering—Toward Therapeutic Drug Monitoring. ACS Sens..

[34] Muneer S., Ayoko G.A., Islam N., Izake E.L. (2020). Utilizing the thiol chemistry of biomolecules for the rapid determination of anti-TNF-α drug in blood. Talanta.

[35] Balčytis A., Ryu M., Seniutinas G., Juodkazytė J., Cowie B.C., Stoddart P.R., Zamengo M., Morikawa J., Juodkazis S. (2015). Black-CuO: Surface-enhanced Raman scattering and infrared properties. Nanoscale.

[36] Muneer S., Ayoko G.A., Islam N., Izake E.L. (2019). Preconcentration and SERS-based determination of infliximab in blood by using a TNF-α-modified gold-coated copper oxide nanomaterial. Mikrochim. Acta.

