# Supplementary material for: Rapid Assay for the Therapeutic Drug Monitoring of Edoxaban
Source: Biomolecules. 2022 Apr 17;12(4):590. doi: 10.3390/biom12040590 (PMC9027065; doi:10.3390/biom12040590)
Supplement: Supplementary file 1 [file biomolecules-12-00590-s001.zip › biomolecules-1677310-supplementary-final.pdf]

Supplementary Material

Rapid assay for the therapeutic drug monitoring of edoxaban

Md Abdur Rashid,[1*] Saiqa Muneer,[2] Yahya Alhamhoom,[1] and Nazrul Islam[3],

[1]Department of Pharmaceutics, College of Pharmacy, King Khalid University, Guraiger, Abha 62529, Kingdom of Saudi Arabia.

[2]School of Chemistry and Physics, Faculty of Science, Queensland University of Technology, Brisbane, 4000, Queensland, Australia.

[3]Pharmacy Discipline, School of Clinical Sciences, Faculty of Health, Queensland University of Technology, Brisbane, 4000, Queensland, Australia.

Corresponding author: Md Abdur Rashid: mdrashid@kku.edu.sa

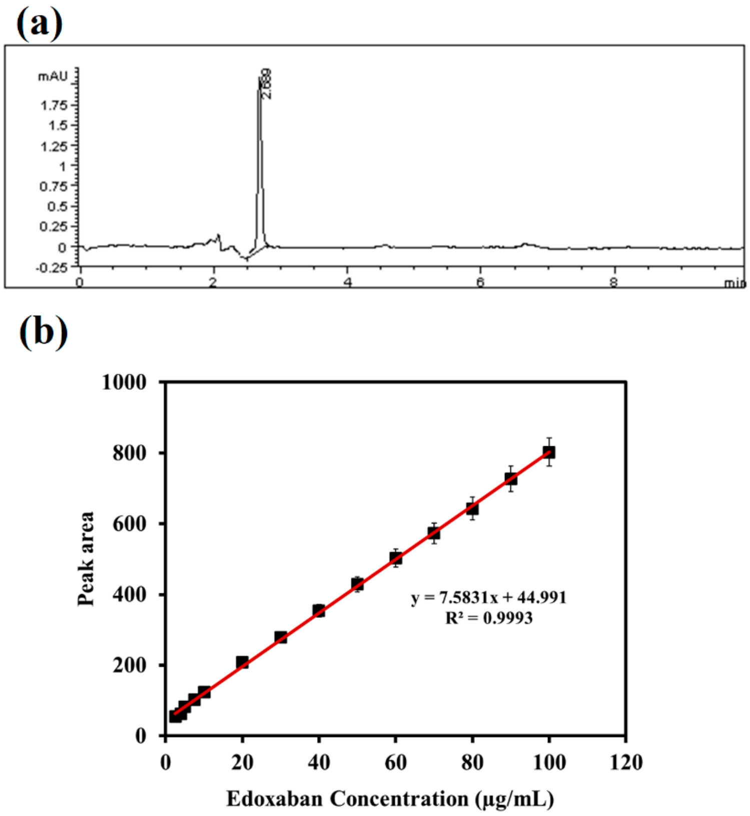

Fig. S1 (a) Chromatographic separation of edoxaban by HPLC-UV method (b) calibration plot of the drug at retention time of 2.68 min.

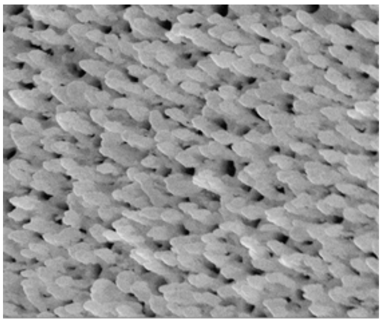

Fig. S2 SEM image of the gold coated silicon nanopillar substrate

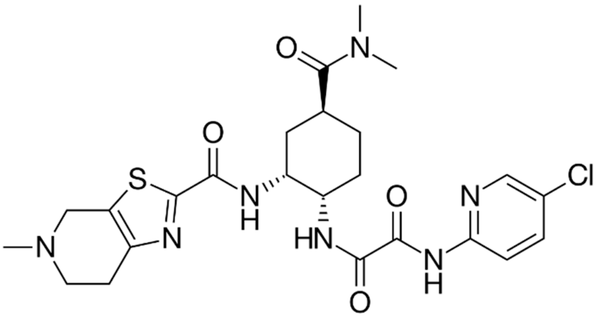

Fig S3. Chemical structure of edoxaban

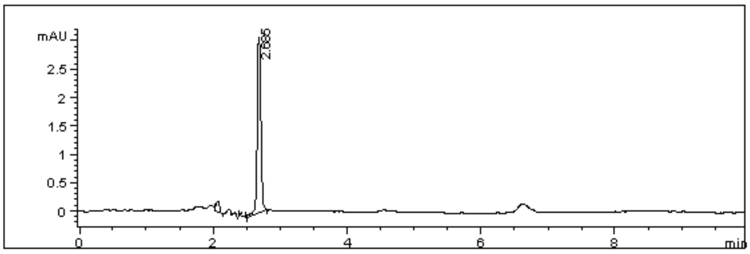

Fig. S4. Chromatographic separation of edoxaban by HPLC-SERS method (retention time of the drug = 2.68 min)
